# Distinct epigenomes in CD4^+^ T cells of newborns, middle-ages and centenarians

**DOI:** 10.1038/srep38411

**Published:** 2016-12-05

**Authors:** Ming Zhao, Jian Qin, Hanqi Yin, Yixin Tan, Wei Liao, Qian Liu, Shuangyan Luo, Min He, Gongping Liang, Yajing Shi, Qing Zhang, Wenjun Cai, Guangliang Yin, Yin Zhou, Jing Wang, Mengying Li, Yi Huang, Aiyun Liu, Haijing Wu, Zhiyong Zhang, Qianjin Lu

**Affiliations:** 1 Department of Dermatology, The Second Xiangya Hospital, Central South University, Hunan Key Laboratory of Medical Epigenomics, Changsha 410011, PR. China; 2 Public Health College of Guangxi Medical University, Nanning 530021, PR. China; 3 State Key Laboratory of Biocontrol, Sun Yat-sen University, Guangzhou 510275, PR. China; 4 Beijing Genomics Institute at Shenzhen, Shenzhen 518083, P.R. China

## Abstract

Age-related variations in genes and microRNAs expression and DNA methylation have been reported respectively; however, their interactions during aging are unclear. We therefore investigated alterations in the transcriptomes, miRNAomes and DNA methylomes in the same CD4^+^T cells from newborn (NB), middle-aged (MA) and long-lived (LL) individuals to elucidate the molecular changes and their interactions. A total 659 genes showed significantly expression changes across NB, MA and LL individuals, in which we identified four age-related co-expression modules with three hub networks of co-expressed genes and non-coding RNAs. Moreover, we identified 9835 differentially methylated regions (DMRs) including 7015 hypermethylated and 2820 hypomethylated DMRs in the NB compared with the MA, and 12,362 DMRs including 4809 hypermethylated and 7553 hypomethylated DMRs in the MA compared with the LL. The integrated analysis revealed a potential relationship between genes transcription and DNA methylation for many age- or immune-related genes, suggesting that DNA methylation-dependent transcription regulation is involved in development and functions of T cells during aging. Our results reveals age-related transcription and methylation changes and their interactions in human T cells from the cradle to the grave. Longitudinal work is required to establish the relationship between identified age-associated genes/DNA methylation and T cells aging phenotypes.

It is becoming clear that epigenetic information is only partially stable and changes across the lifespan of an organism, representing a drawbridge between genetics and environment^1^. Most studies have demonstrated that aging is associated with a relaxation of epigenetic control; on the one hand, a decrease in global cytosine methylation has been observed during aging, mostly because of demethylation in transposable repetitive elements^2^,^3^,^4^. On the other hand, age-related methylation alterations have been observed in promoter regulatory regions of specific genes belonging to some biological processes, such as cell cycle regulation, tumor cell invasion, apoptosis, metabolism, cell signaling and DNA repair^5^,^6^,^7^. However, no report to date has yet identified the patterns of DNA methylation, genes and microRNAs (miRNAs) expression and their interactions in the same immune cells during healthy human aging.

Epidemiological studies have revealed that long-lived individuals—people surviving to the 95th percentile of the respective birth cohort-specific age distributions—frequently present a healthy course of the aging process, with the absence or a delayed onset of age-related diseases^8^,^9^. Hence, long-lived individuals may offer a key to elucidate the molecular mechanisms underlying the healthy aging phenotype. To characterize healthy aging immune-related mechanisms at different stages of human aging, we selected subjects from the Bama County of China, which contains a high centenarian proportion and is known as the “Longevity County”^10^. The healthy elders with extreme longevity involved in our study may increase our understanding of aging and longevity. We hypothesized that a systematic analysis of the profiles of mRNA and miRNA expression and DNA methylation in individuals at different age stages would help to identify key alterations during human natural aging. Thus, we performed a genome-wide methylation analysis in newborn (NB), middle-aged (MA) and long-lived (LL) individuals using methylated DNA immunoprecipitation (MeDIP)-sequencing and combined these results with mRNA and miRNA sequencing (RNA-seq and miRNA-seq) data. Our data revealed dynamic changes in networks of gene expression and epigenetic regulation in T cells during healthy human aging and provide pivotal information to help further understand age-related diseases.

## Results and Discussion

### Age-related transcriptome analysis in CD4^+^ T cells

**Figure 1:**
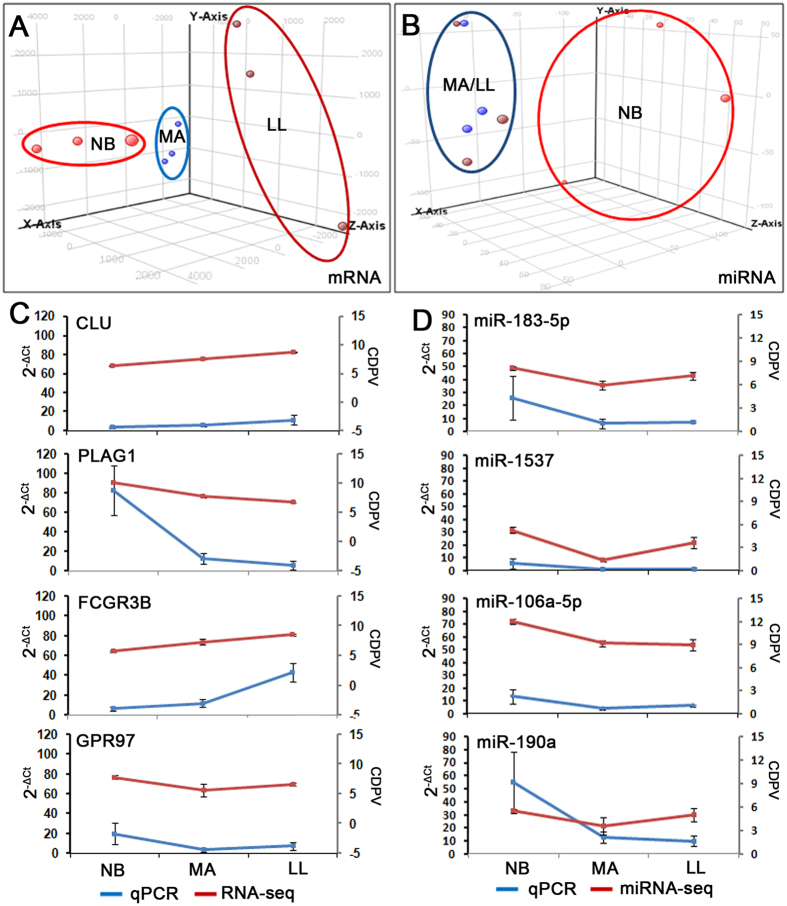
Global analyses of the miRNAomes and transcriptomes of human CD4^+^ T cells during the aging process. (**A**) Stage-transitive transcriptome changes of human CD4^+^ T cells. (**B**) Stage-transitive miRNAome changes of human CD4^+^ T cells. Human CD4^+^ T cell samples are projected onto the three-dimensional space captured by PCA. Each of the staged aging samples is colored as indicated. (**C,D**) Validation of sequencing data by quantitative RT-PCR of known genes and miRNAs in CD4^+^ T cells. Experiments were performed using residual CD4^+^ T cell RNAs used for the RNA-seq. Data are represented as the means ± SDs.

To investigate the patterns of changes of gene expression in chronological healthy aging, we collected peripheral blood from three age stages, including NB, MA and LL, in “Bama” county, China. Three biological replicates were collected for CD4^+^ T cells at NB, MA and LL; because of practical limitations and material availability, every thirty peripheral blood samples at each age stage were pooled together and immediately subjected to CD4^+^ T cells isolation and RNA extraction. We included 90 individuals from each age stage, distributed equally into 3 pools with identical male/female ratios. CD4^+^ T cells were isolated and subjected to RNA-seq with three biological replicates. After data normalization as described in the Materials and Methods, Principal Component Analysis (PCA) indicated that the 9 sample pools were more distinct in NB compared with the other two stages, suggesting that a major transition occurred among NB, MA and LL (Fig. 1A and B). There were 1685 up-regulated and 1675 down-regulated mRNAs and 50 up-regulated and 46 down-regulated miRNAs between the MA and NB groups. Three hundred and one up-regulated and 164 down-regulated mRNAs and 6 up-regulated and 11 down-regulated miRNA were also identified between the LL and MA groups, suggesting that the transcriptome and miRNAome were chiefly altered during the NB to MA transition (Supplementary Fig. S1). Independently, quantitative RT-PCR (RT-qPCR) was employed to validate genes and miRNA expression patterns that were connectively altered from NB to LL in the pooled samples used in RNA-seq and un-pooled samples, indicating that these candidates behaved as expected (Fig. 1C and D, Supplementary Fig. S2A and S2B).

**Figure 2:**
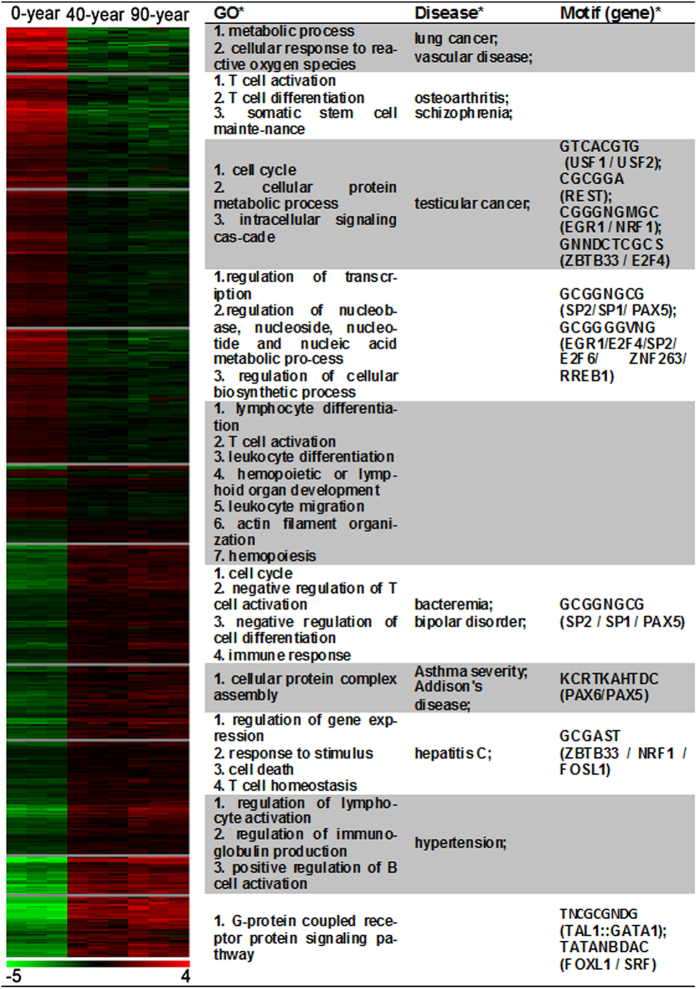
In-depth analyses of transcriptome features characterizing CD4^+^ T cell during the aging process. Illustration of gene expression patterns and corresponding biological theme enrichments for each of the ten gene clusters. The patterns of gene expression in 0-year (NB), 40-year (MA) and 90-year (LL) with green to red blocks, symbolizing relative expression levels corresponding to the scale bar at the bottom. Various biological annotations were mined to determine enrichments of biological relevance, as highlighted by GO analysis for functional enrichments (P-values < 0.05), motifs of JASPAR conserved transcription factor binding sites for regulatory enrichments (P-values < 0.05), and Online Mendelian Inheritance in Man (OMIM) disorder-gene association information for disorder enrichments (P-values < 0.05).

Next, an extraction of differential gene expression-based methodology, the one-way analysis of variance (ANOVA) test, was applied to identify mRNAs with differential expression patterns that were consistent between replicates but differentially regulated across the various aging stages. The resulting matrix contained expression measurements for 659 candidates across 9 pooled samples, denoted as the human aging expression matrix (hAEM) (Supplementary Table S1), and was used for the subsequent analyses. In an attempt to identify the transcriptome features inherent to the hAEM, we first combined k-means clustering with expression measurements of the 659 candidates across the 9 sample pools, and then subjected to a gene clustering procedure that revealed 10 clusters based on the Euclidean metric (termed clusters 1–10). Genes within each of the 10 clusters displayed highly similar expression patterns (left panel of Fig. 2), suggesting that they may share common features. In addition, the expression patterns of genes in clusters 1, 2, 3, and 4 displayed overall similarity, with gene expression levels tending to be gradually repressed as aging progressed, whereas the expression levels of genes in clusters 6, 7, 8, 9 and 10 gradually increased.

We performed enrichment analysis of the genes in these clusters to examine whether they shared functional or regulatory features using Gene Ontology (GO) and the JASPAR conserved transcription factor-binding sites (TFBSs)^11^. We observed a congruent relationship between the common promoter motifs and conserved TFBSs. As shown in the right panel of Fig. 2, significant GO terms in clusters 1–4 included those associated with metabolism, T cell activation, cell cycle and transcription regulation. Only a few significant TFBSs were observed, including apoptosis- and DNA damage-related transcription factors, such as EGR1^12^ and E2F4^13^. The most significant GO terms in clusters 6–10 were more similar, representing a wide spectrum of functions involved in the negative regulation of T cell activation, the cell cycle and gene expression regulation. The most significant TFBSs included cell proliferation, differentiation, and transformation regulators, such as FOSL1^14^, SRF^15^ and GATA1^16^.

Finally, we used the disorder-gene association information from the Online Mendelian Inheritance in Man (OMIM) to conduct enrichment analysis of the genes in the clusters. As illustrated in the middle panel of Fig. 2, significant phenotypes related to gene clusters 1–4 were mostly associated with age-related diseases, including cancer, vascular disease and osteoarthritis, whereas the genes in clusters 6–10 were primarily linked to pathogen infection and asthma.

### Identification of a CD4^+^ T cell aging-specific co-expression module

**Figure 3:**
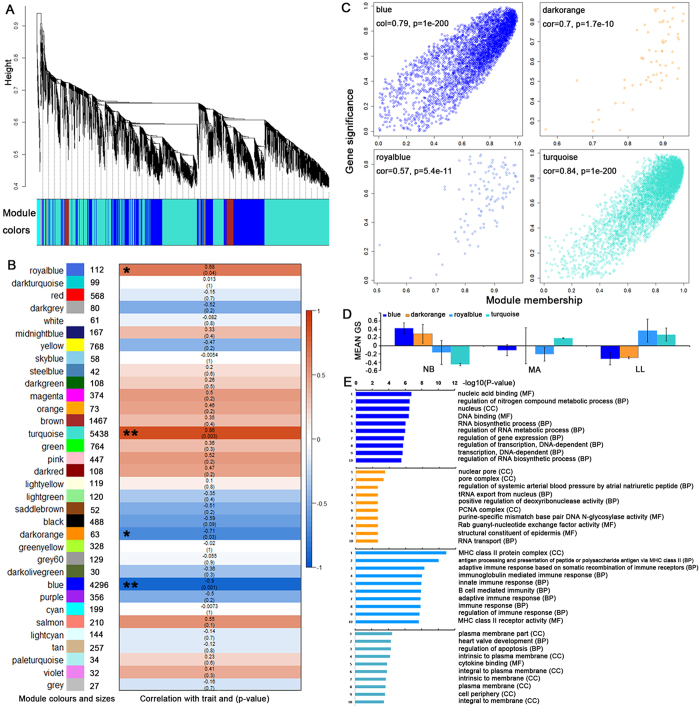
Module identification in CD4^+^ T cell samples and correlations of module expression with aging. (**A**) Clustering dendrogram of genes indicating module membership in colors. (**B**) Module sizes and correlation of module eigengenes with stages of aging. Module-wise correlations are provided along with the P-values of the correlation in cells colored by the strengths of the correlation. Modules significantly associated with the trait (absolute correlation > 0.5 and P-value < 0.05) are indicated with an asterisk (*). (**C**) Scatterplots of gene significance versus module membership for aging-associated modules, with correlations and P-values indicated. (**D**) Average ‘gene significance’ (GS) of genes within a specific module are summarized in the barplot for each aging stage (left to right: NB, MA, and LL). The blue and dark orange modules are positively associated with NB. The royal blue module is positively associated solely with LL. The turquoise module is negatively associated with NB. (**E**) The top ten GO terms were calculated with the models by functional enrichment (P-value < 0.05).

To further identify a hierarchical network view of co-expressed genes across aging subtypes, we applied WGCNA to a dataset consisting of 3 NB, 3 MA and 3 LL specimens^17^. Unsupervised average linkage hierarchical clustering of all candidates (including protein coding genes, lncRNAs and miRNAs) in this dataset resulted in 34 modules (Fig. 3A and B) labeled by color, and each consisted of mutually exclusive co-expressed candidates. Candidates with no distinct module assignment were grouped in a grey module by WGCNA. Four of these modules, including blue, royal blue, dark orange and turquoise, were identified using any pre-assigned phenotype (GS P-value < 0.05). Figure 3B depicts the module sizes and correlations of the module eigengenes to the trait. The module membership (MM) versus gene significance (GS) plots for these modules (Fig. 3C) demonstrated that MM and GS were highly correlated, indicating that candidates most significantly associated with the trait were often also the most important (i.e., central) elements of the respective modules. Following the unsupervised module generation, individual candidate correlations to a specific aging-stage were quantified by GS. The average GS of all candidates within each module is summarized in Fig. 3D. This analysis unveiled positive or negative correlations of certain modules with specific aging stages (Supplementary Table S2). The blue module contained candidates positively correlated with the NB stage, making it an “infant module” that included the regulation of gene expression and RNA metabolic terms from GO enrichment analysis (Fig. 3E). The dark orange module contained candidates positively correlated with the NB stage and negatively correlated with the LL stage, making it a “mix module” that included the PCNA complex and RNA transport terms (Fig. 3E). The royal blue module contained candidates positively correlated to the LL stage, making it an “elder module” that included immune response terms (Fig. 3E). The turquoise module contained candidates positively correlated with the MA and LL stages and negatively correlated with the NB stage, making it an “adult module” that included apoptosis regulation and development-related terms (Fig. 3E).

### Identification and validation of an aging-specific hub network of co-expressed genes across the transcriptomic and miRNAomic platforms

**Figure 4:**
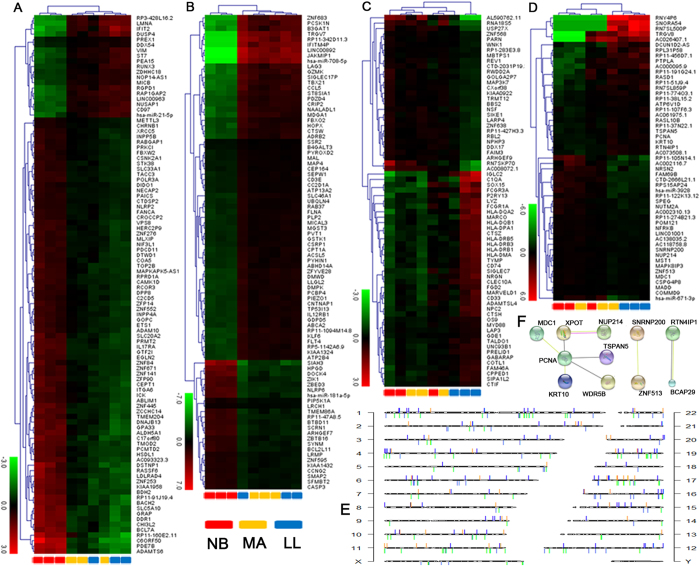
Validation of WGCNA models as robust classifiers for age-specific stages in four independent model datasets from the blue, turquoise, royal blue and dark orange modules. (**A**) Unsupervised clustering heatmap based on the top 99 transcripts and one miRNA (rows) of CD4^+^ T cells samples (columns) in the blue model dataset. Red and green indicate high and low expression levels, respectively. All of NB group clusters by themselves on the far left of the dendrogram. (**B**) Unsupervised clustering heatmap based on the top 86 transcripts and two miRNAs of CD4^+^ T cells samples in the turquoise model dataset. All of NB group clusters by themselves on the far left of the dendrogram. (**C**) Unsupervised clustering heatmap based on the top 69 transcripts of CD4^+^ T cells samples in the royal blue model dataset. None of the age-specific groups cluster by themselves on the dendrogram. (**D**) Unsupervised clustering heatmap based on the top 51 transcripts and two miRNAs of CD4^+^ T cells samples in the dark orange model dataset. All of LL group clusters by themselves on the far right of the dendrogram. Red and green symbolizing relative expression levels corresponding to the scale bar at the bottom left for (**A**–**D**). (**E**) Locations of the model-related transcripts and miRNAs (Model-RNAs). Transcripts and miRNAs were mapped to corresponding genomic coordinates on human chromosomes and are represented on an Ensemble-generated ideogram (Ensemble, hg19 genome build). Model-RNAs are colored in blue, turquoise, royal blue and dark orange, corresponding to their models. (**F**) A connected network illustrates Model-RNAs in the dark orange model that contained genes positively correlated to the age-specific phenotype. Layouts of the connected network are based on correlation information annotated in the STRING database.

To identify a network of co-expressed candidates that was specific to aging, we focused on the blue, turquoise, royal blue and dark orange modules as described above. In gene co-expression networks, connectivity measures how correlated a candidate is with all other network candidates. Because the blue and turquoise modules were rather large, we could restrict the output candidates to remain the top hub candidates in these four modules with a connectivity threshold of 0.3 and a gene number of 100. As a result, each module consisted of a few highly connected “hubs” (genes that have high intramodular connectivity kME) and many candidates with fewer connections. To validate the robustness of the co-expression network as an aging-specific classifier, we first applied unsupervised hierarchical clustering bootstrap analysis to the expression value of each aging sample in a test dataset from which the blue, turquoise, royal blue and dark orange modules were derived. The NB-specific blue module (100 candidates; Fig. 4A) consisted of many more up-regulated candidates across the NB stage. In contrast, the turquoise module (88 candidates; Fig. 4B) consisted of many more co-expressed down-regulated candidates across the NB stage. The majority of candidates within the royal blue modules (69 candidates; Fig. 4C) positively associated with the LL stage but had a nonspecific cluster across LL, MA or NB stages and thus was excluded from further analysis. The dark orange module (53 candidates; Fig. 4D), containing most candidates clustered across the LL stage, did not cluster across the NB stage, suggesting that candidates in this module behaved as expected to the LL characteristic. Candidates in dark orange module were distributed on genome randomly act as effective genetic markers as well as other modules (Fig. 4E), suggesting that they did not have a chromosome preference.

Because genes do not function in isolation, we investigated the interconnectedness of the dark orange module (elder module) in the STRING molecular network database to identify biological processes, such as RNA transport. The STRING tool integrates interaction information to identify the function-related subnetworks^18^. As a result, we obtained a subnetwork specific for the dark orange module (Fig. 4F). As gene expression in the PCNA-centered subnetwork was present no significant differential change between NB vs MA and LL vs MA (P-value < 0.05, FC > |2|), whereas the PCNA complex-related genes experienced gradually altered dynamics from NB to LL, suggesting that DNA repair mechanism activation in response to DNA damage is an important characteristic in human aging process.

**Figure 5:**
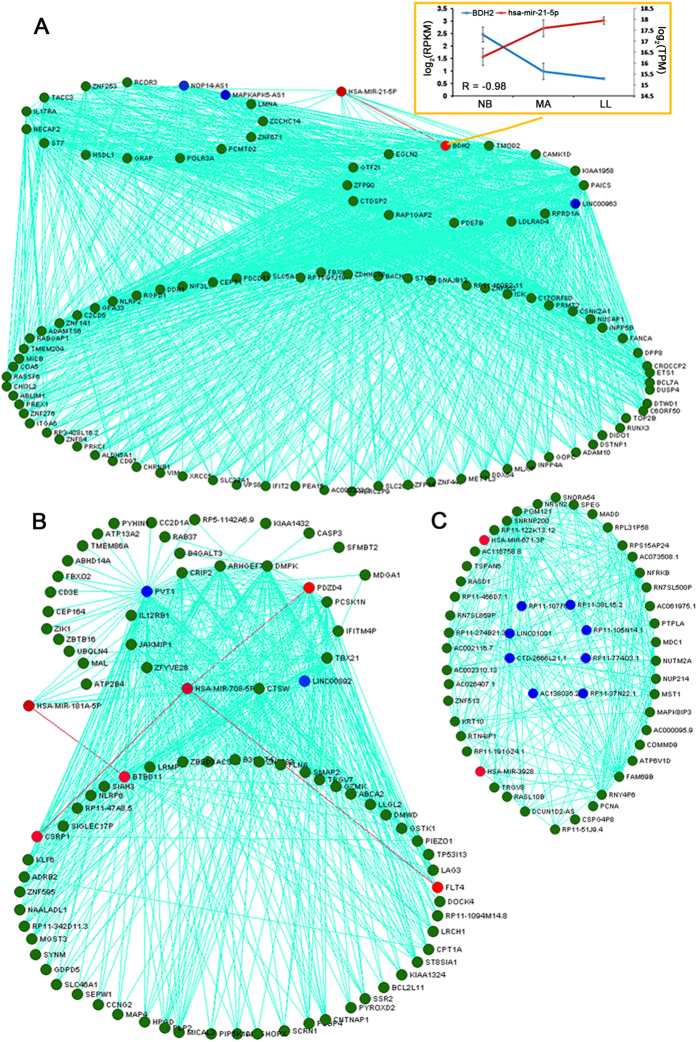
Visualization of the networks of selected modules associated with the aging process. Totals of 100 genes in the blue module, 88 in the turquoise module and 53 in the dark orange module are depicted in networks (**A**,**B** and **C**) respectively. Nodes in the network are labeled by their gene symbols. Each node is colored based on RNA type (blue representing lncRNA, red representing miRNA and green representing coding RNA). To improve network visibility, the edges (connections) have been filtered to display only those with correlation weights above 0.5 in the turquoise, 0.3 in the dark orange and 0.45 in the blue modules. The predicted target genes of miRNAs in each network are connected with red edges, of which had high coefficient of correlation between miRNA and gene expression are displayed at the blue module of the network with yellow box.

These top candidates were also among the most well-connected RNAs in the network visualizations constructed using the refined set of candidate RNAs within the blue (Fig. 5A), turquoise (Fig. 5B) and dark orange modules (Fig. 5C). The results demonstrated that infant modules were closely related with transcription factor genes, which indicated that the module and genes might synergistically cause genes transcription. For instance, BACH2 was associated with cell cycle and transcriptional regulation in response to DNA damage and immune cell aging^19^,^20^. Runx3 deficiency can result in myeloproliferative disorders in aged mice^21^. These transcription factors are related to aging progress. Notably, BDH2 is one of the predicted target genes of miR-21–5p, which has been previously identified to regulate mitochondrial iron homeostasis^22^. Mitochondrial dysfunctional has been widely reported with aging^23^. Previous study also shown many mitochondrial ribosomal genes expression were changed in CD14^+^ monocytes during aging^24^. Both BDH2 and miR-21-5p not only have a high connectivity in our co-expression network, but also appearing in an obvious negative correlation (R = −0.98, P < 0. 05) for expression dynamics (Fig. 5A). Therefore, our dataset constructs a potential bridge between miR-21 and mitochondrial dysfunction in T cells through regulating BDH2 expression and suggests that the expression of miR-21 and BDH2 may be a candidate marker of human aging.

The results showed that adult modules were closely related with heart development and apoptosis-related genes, which indicated that the module and genes might synergistically cause CD4^+^ T cell metabolism. For instance, TP53-inducible genes play crucial roles in many biological processes, including cell cycle control, DNA repair, and apoptosis. DMPK is a stress-inducible gene in both a TP53-dependent and independent manner and has been implicated in cardiac and behavioral dysfunctions^25^. Myotube loss and atrophy of 15-day-differentiated DM1 myotubes (DMPK is the protein product of the human DM1 locus on chromosome 19q13.1) indicated activated catabolic pathways, as confirmed by caspase-3 activation (CASP3), cytochrome c release, and chromatin fragmentation^26^. The high connectivity between DMPK and CASP3 within our co-expression network (Fig. 5B) is consistent with previous observations. Herein, we identified other TP53-inducible candidates, such as TP53i13 (damage stimulated cytoplasmic protein 1, DSCP1)^27^, non-protein coding RNA PVT1^28^ and miR-181a/b^29^. These candidates were connected together with high connectivity, suggesting that TP53 may regulate immunosenescence as discovered by previous studies^30^,^31^. Our result showed that long noncoding (lnc)RNA PVT1 is centrally involved in adult model, suggesting that this non-protein coding RNA may play an important role in diverse pathways during aging process.

The results demonstrated that dark orange module was closely related to RNA transport, which suggests that this module and genes might synergistically cause protein translation. Surprisingly, the majority of candidates with high connectivity in the co-expression network were lncRNAs or pseudogenes, suggesting that non-coding RNAs might also play an important roles in T cells during human aging process.

### An overview of the age-related methylome in CD4^+^ T cells

**Figure 6:**
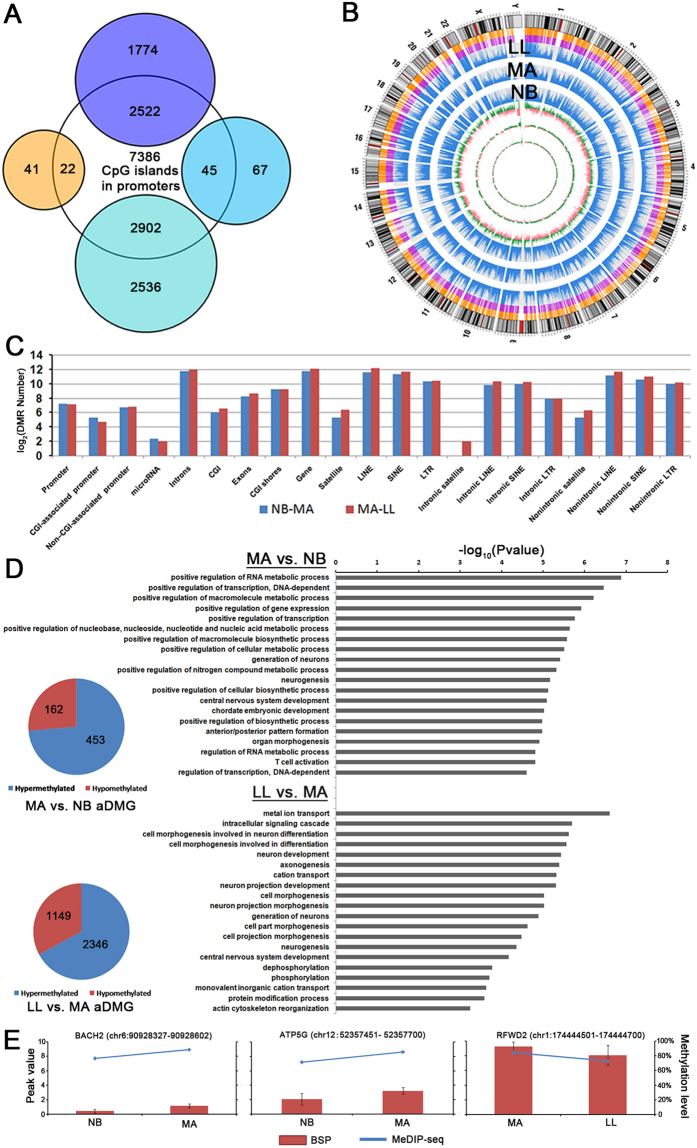
Age-specific differentially methylated regions (aDMRs) and related genes (aDMGs). (**A**) Venn diagram of the distribution of genes corresponding to the blue, turquoise, royal blue and dark orange modules based on whether they possess CpG islands in promoters. (**B**) Circos representation of genome-wide DNA methylation levels in the NB, MA, and LL groups. From outer to inner, this graph displays chromosomal karyotypes; promoters; CpG islands; read distributions of the LL, MA, NB groups in 100-kb segments; and differential DMR distributions of NB vs MA, MA vs LL and NB vs LL. Green represents log_2_ ratios > 0, and red represents log_2_ ratios < 0. (**C**) aDMR distribution among different genomic sequences. (**D**) aDMG distribution according to the direction of the DNA methylation change, and GO enrichment analysis of these aDMGs between MA and NB individuals and between LL and MA individuals. (**E**) The patterns of change in promoter methylation of three aDMGs (BACH2, ATP5G and RFWD2) confirmed by BSP are consistent with the results from MeDIP-seq.

To identify gene expression potentially influenced by DNA methylation, we used bioinformatics tools to determine whether CpG islands existed in the regulatory elements of the genes in the blue, dark orange, turquoise and royal blue modules. We defined the regulatory elements as sequences from 2,000 bp upstream to 500 bp downstream of the transcription start sites. The results indicated that 2522 (58.7%) of the 4296 candidates in the blue module, 45 (40.2%) of the 112 candidates in the royal blue module, 2902 (53.3%) of the 5438 candidates in the turquoise module, and 22 (34.9%) of the 63 candidates in the dark orange module contained CpG islands (Fig. 6A). These results did not include the remaining candidates (including ncRNAs) within either intron or body regions, implying that methylation may contribute to the expression of most candidates in T cell during human aging process. Thus, we obtained genomic DNA from the aforementioned CD4^+^ T cells of three separate pools at three age groups and generated the corresponding methylomes in triplicates for each age group using MeDIP-seq. After data filtering as described in the Materials and Methods, we obtained averages of 64% (SD ± 6.25%), 65% (SD ± 1.17%) and 71% (SD ± 1.41%) of high-quality unique mapping data per group for further analysis. Approximately 35% the human genome was covered at least 1-fold. The reads of MeDIP-seq covered most of the CpG islands (CGIs) and promoter regions in genome-wide. Locations of CGIs, the reads in 100-kb segments, and the log2-transformed average-fold changes among the NB, MA and LL groups for the reads are plotted for the each chromosome in entire genome using Circos (Fig. 6B).

Next, the MACS pipeline was used to determine age-specific differentially methylated regions (aDMRs) between the different age groups. Based on reads coverage, we performed pairwise comparisons among the three groups to identify aDMRs. We identified 9835 DMRs including 7015 hypermethylated and 2820 hypomethylated DMRs in the NB group compared with the MA group (nmDMRs), and 12,362 DMRs including 4809 hypermethylated and 7553 hypomethylated DMRs in the MA group compared with the LL group (mlDMRs) (Supplementary Table S3 and Table S4). The cut-off of DMRs is ≥1.5 fold and P-value < 0.001.

We subsequently mapped reads to their nearest genomic feature and performed an enrichment analysis on all DMRs in different genomic regions, including promoter, CGI-associated promoter, non-CGI-associated promoter, microRNA, introns, exons, CGI shore, gene and repeat sequences families including satellites, short interspersed nuclear elements (SINEs), long interspersed nuclear elements (LINEs) and long terminal repeats (LTRs). By comparing the number of enrichments in different genomic elements in both NB vs MA and MA vs LL (Fig. 6C), we observed that many more hyper- or hypo-methylated DMRs were located in intron and gene body. Regarding repeat elements, we observed that many more hypo- and hyper-DMRs existed in SINEs, LTRs and LINEs between two groups, which were similar to results from previous studies in cancer^32^.

### Identification of genes with DMRs

To further study the DNA methylation status at certain genomic regions, the genomic loci that corresponded to DMRs were associated with genes according to their relative positions in the human genome, including 2 kb upstream of transcription starting site (Upstream2k), 5′-untranslated region (5′-UTR), CDS, 3′-UTR and 2 kb downstream of transcription ending site (downstream2k) of gene. An amount of 5% of the uniquely mappable reads in genes according to their relative position had a minimum of 10 reads that allowed us to confidently detect the presence of aDMRs among the NB, MA and LL groups. A total of 636 significant (FC > 2, FDR < 0.05) aDMRs were identified between MA and NB, of which 464 were hypermethylated and 171 hypomethylated in the MA samples. A total of 3595 significant aDMRs were identified between MA and LL, of which 2417 were hypermethylated and 1178 hypomethylated in the LL samples (Supplementary Table S5).

Furthermore, 615 aDMRs were in the upstream 2 k, 5′-UTR or gene body between MA and NB, represented by age-specific differentially methylated genes (aDMGs). Three thousand four hundred ninety-five of DMRs were in the upstream 2 k, 5′-UTR or gene body between MA and LL (Fig. 6D, left panel). To assess whether the genes with DMRs in the upstream 2 k, 5′-UTR or gene body were enriched for certain biological processes or pathways, we performed GO analysis using the DAVID Bioinformatic Database (DAVID – Database for Annotation, Visualization, and Integrated Discovery). This analysis indicated that the top 20 significant GO terms, including regulation of transcription, RNA metabolic process and T cell activation, were enriched in genes with methylation regulation in the MA group compared with the NB group. However, excretion, metal ion transport, neuron development and differentiation were enriched significantly in genes with methylation regulation in the LL group compared with the MA group (Fig. 6D, right panel). Finally, we selected three aDMGs, including BACH2 (hyper-methylation between NB and MA), ATP5G2 (hyper-methylation between NB and MA), and RFWD2 (hypo-methylation between MA and LL) to validate their promoter methylation status in the same samples and un-pooled samples by bisulfite sequencing (BSP) (Fig. 6E and Supplementary Fig. S3).

### Integrated analysis of DNA methylation and gene expression profiles during human natural aging

**Figure 7:**
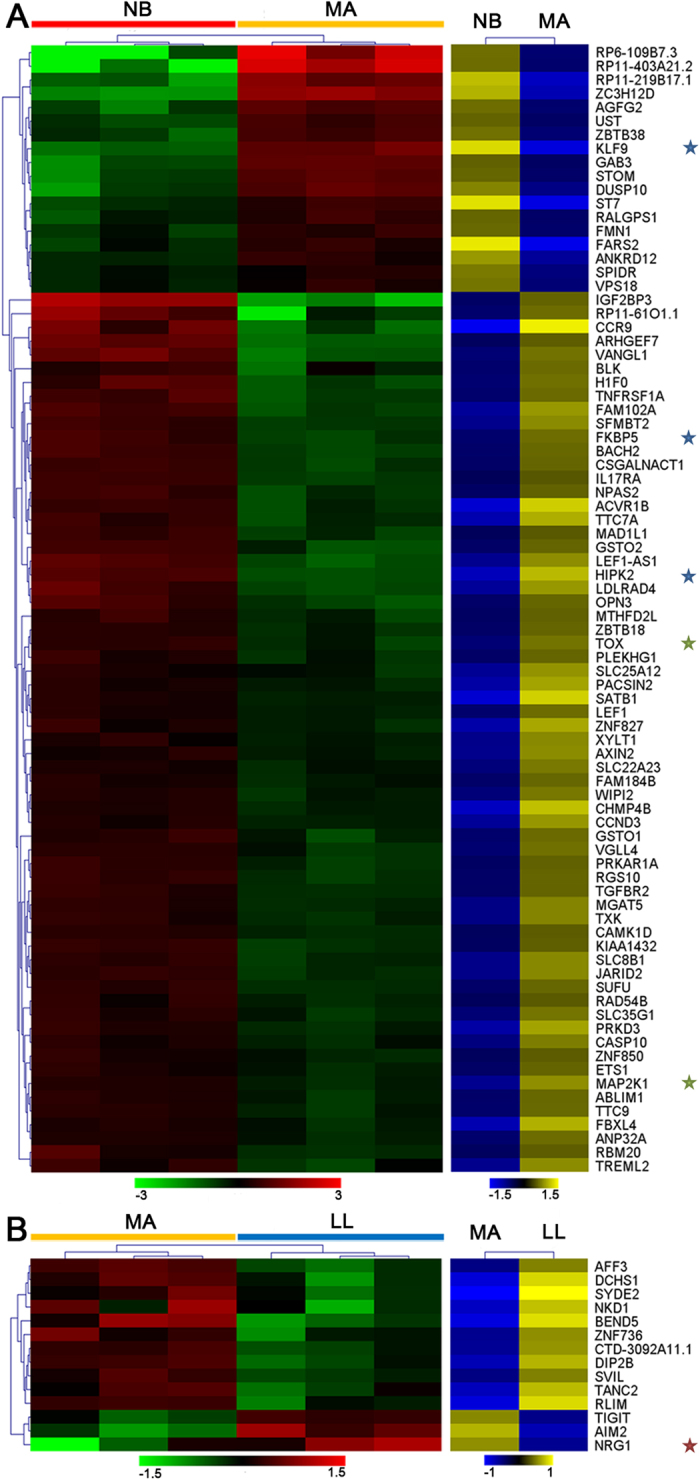
Integrated analysis of DMGs and differentially expressed genes (DEGs) in the database of WGCNA models. Unsupervised hierarchical clustering heatmap of hypermethylated and down-regulated genes or hypomethylated and up-regulated genes across CD4^+^ T cell samples distinguished MA from NB individuals (**A**) and LL from MA individuals (**B**). Red and green indicate high and low expression, and yellow and blue indicate hyper- and hypo-methylation, respectively. Gene symbols are indicated on the right. Red, blue and green pentagrams represent genes within the GenAge, GenDR and longevityMap from HAGR databases, respectively. Red and green represent relative expression levels corresponding to the scale bar at the bottom, yellow and blue represent relative methylation levels corresponding to the scale bar at the bottom.

Hypomethylation in regions rich in CpG islands produces active transcription, whereas DNA hypermethylation reduces gene transcription^33^. Previous study has shown that DNA methylation profiles were negatively or positively correlated with age-associated gene expression profiles in CD14^+^ monocytes during aging process^5^,^24^. Here, we performed an integrated analysis of DNA methylation and gene expression of genes in blue, dark orange and turquoise modules. Genes with aDMRs within upstream 2 k, 5′-UTR and gene body were selected and coupled with differential gene expression. As shown in Fig. 7, we identified 82 simultaneously differentially methylated (FC > |1.5|, FDR < 0.05) and expressed genes (FC > |1.5|, FDR < 0.05) between the NB and MA groups and 14 genes between the MA and LL groups. Furthermore, we filtered these data through the Human Ageing Genomic Resources (HAGR)^34^ database and identified aging-associated genes, such as NRG1 within the GenAge database, KLF9, FKBP5 and HIPK2 within the GenDR database, and TOX and MAP2K1 within the longevityMap database.

Notably, we observed an increased DNA methylation level at the BACH2 locus and decreased BACH2 expression in CD4^+^ T cells of the MA and LL groups compared with the NB group (Fig. 7B and Supplementary Fig. S4). BACH2 plays a crucial role in T-cell-mediated immune responses^35^. A recent study by Kuwahara *et al*. demonstrated that Menin binds to the BACH2 locus and controls BACH2 expression through the maintenance of histone acetylation. Kuwahara *et al*. also observed decreased Menin binding and BACH2 expression in senescent mouse CD4^+^ T cells^20^. This finding suggests that BACH2 down-regulation may be associated with T cell senescence, which may be controlled by DNA hypermethylation at the BACH2 locus besides of histone modifications.

## Conclusions

In conclusion, we identified continually altered patterns of genes and non-coding RNAs transcription and DNA methylation in the T cell during aging process of healthy people. We found some important immune-related genes in T cells that may be markers of human natural aging, which will provide potential targets for studies on age-related diseases. The integrated analysis of transcriptome, miRNAome and methylome data also revealed a potential epigenetic mechanism during aging process that is important for comprehensively understanding the mechanisms by which environmental factors regulate aging and for uncovering the secret of longevity in the Bama county of Guangxi, South of China.

Several limitations of the study should be noted. The samples in this study are total CD4^+^ T cells, which contain these subsets such as naive, memory, effector cells. Because different age contains different proportions of subsets in total CD4^+^ T cells, the comparative analysis in the same subsets of CD4^+^ T cells provide more precise information for understanding aging phenotype and age-related diseases. Secondly, we found some potential association between genes expression/DNA methylation and aging process in T cells, but the longitudinal work is necessary to confirm the relationship between identified age-associated genes/DNA methylation and aging phenotypes or diseases.

## Methods

### Sample preparation

Peripheral blood was obtained from 90 healthy newborn (0 y, NB), 90 healthy middle-aged (age range from 40 to 55 years, MA) and 90 long-lived subjects (age range from 90 to 110 years, LL) living in the Bama county of the Guangxi province of southern China, which is known for its longevity. The NB samples came from umbilical cord blood. All subjects acceded to a basic physical examination and chest X-rays when the blood samples were harvested. Primary criteria for inclusion in the study were the absence of overt systemic diseases, such as cancer, cardiovascular events, autoimmune diseases or dementia. Furthermore, the LL subjects had to be able to perform some daily living activities, and even simple farm work and answer a health and family history questionnaire. Written informed consent was obtained from all study participants or from family representatives if the participants could not provide consent because of reasons such as illiteracy or being newborn. Approval for the project was received from the Ethics Committee of the Second Xiangya Hospital of Central South University and the Guangxi Medical University. All experiments were carried out in accordance with the guidelines and regulations set by the Ethics Committee of the Second Xiangya Hospital of Central South University and the Guangxi Medical University.

Thirty blood samples were pooled in each group according to the female⁄male ratio of 4:1. After pooling, peripheral blood mononuclear cells (PBMCs) were isolated immediately from the pooled blood samples using density gradient centrifugation, and CD4^+^ T cells were isolated by positive selection using CD4 beads according to protocols provided by the manufacturer (Miltenyi, Bergisch Gladbach, Germany). In addition, 20 un-pooled samples including 6 NB samples, 8 MA samples and 6 LL samples were harvested for supplementary validation of gene expression and DNA methylation. The proportions of naïve, memory and regulatory T cells in the un-pooled samples were detected by flow cytometry (Supplementary Table S6).

### Analysis of mRNA expression profile (RNA-seq)

RNA was isolated from the NB, MA and LL samples using RNeasy RNA mini kit (Qiagen) and underwent quality control using Agilent Bioanalyzer 2100 (Agilent). Poly-A library preparation and sequencing were performed following the manufacturer’s protocol (Illumina). The prepared libraries were sequenced using Illumina HiSeq™ 2000 with up to 20 million (M) reads per sample which generated 2 × 100 bp paired-end reads. The saturation analysis indicated that we had sufficient reads to give reproducible genes expression profiles (Supplementary Fig. S5A).

Clean sequencing reads were mapped to the human Ensembl-RNA reference sequence (downloaded from http://asia.ensembl.org/Homo_sapiens/Info/Index) using the FANSe 2 algorithm^36^ with the parameters -L110 -E7 -U0 -S10. Alternative splice variants were merged. Genes with at least 10 mapped reads were considered to be reliably detected genes. These genes were further quantified using count values, which were raw counts of sequencing reads. The count values were imported into DESeq package^37^ of R software (http://www.rproject.org) to calculate the up-/down-regulation of genes among the NB, MA and LL groups, respectively. This software provides methods to produce condition-dependent per-gene values (CDPVs) and differential expression values using the negative binomial distribution and a shrinkage estimator for the distribution’s variance. The up- or down-regulated genes between two groups including NB *vs* MA or MA *vs* LL were identified by filtering the RNA-seq data with the following cut-off: 1.5-fold change in expression levels and P-value < 0.05. Furthermore, GeneSpring software (Agilent) was used to identify the genes with continuously changed expression among NB, MA and LL using one-way ANOVA tests. Multiple testing corrections were performed using Benjamini-Hochberg with a more stringent threshold P-value < 0.01. One-way ANOVA analysis of temporal transcription profile could only reveal the significantly changed genes deviate from at least one of the three groups, and therefore these genes with continuously changed expression from NB to LL were further filtered with 1.2-fold ratio. Dynamically regulated gene expression patterns were clustered using the k-means algorithm in GeneSpring. Common promoter motifs from 5000 bp upstream of the genes included in each k-means cluster were extracted using the BCRANK package.

Gene expression was quantified as reads per kilobase of candidates per million mapped reads (RPKM), calculated by dividing CDPV by the respective gene lengths expressed in kilobase pairs. The gene lengths were taken as the length of the longest transcript for the respective genes obtained from the Ensembl-RNA reference sequence. CDPV values were used for the different expression (DE) analysis, and RPKM values were used for the co-expression analysis. Clustering of samples by gene expression data (transformed to log2 CDPV) was evaluated using principle component analysis (PCA) function in GeneSpring.

### Analysis of miRNA expression profiles

Total RNA samples were prepared for miRNA sequencing using Illumina’s Small RNA v1.5 Sample Preparation Guide and subjected to 50 bp single end (SE) sequencing on the Illumina GA IIx platform. Low-quality reads were trimmed, and adapter sequences were accurately clipped with the aid of a dynamic programming algorithm before subsequent statistical analysis. The leading 18 bases were trimmed from the 50-bp reads based on the quality score and the length of mature miRNAs. The trimmed reads from the 9 pooled samples were mapped to miRNA precursor sequences (downloaded from miRBase 19.0) using the Bowtie software^38^, respectively. No differences between the reads and the miRNA precursor sequences were allowed, which indicates exact matches only. The number of reads mapped to a miRNA sequence was taken to represent the expression level of that particular miRNA. The same pipeline used for mRNA differential expression analysis was also used for miRNA expression analysis. The saturation analysis indicated that we had sufficient reads to give reproducible genome-wide miRNAs expression profiles. (Supplementary Fig. S5B).

miRNA expression was also quantified as tags per million (TPM), calculated by dividing CDPV by the respective miRNA reads number expressed in kilobase pairs. CDPV values were used for the DE analysis, whereas TPM values together with RPKM values as described above were used for the co-expression analysis. The clustering of samples by miRNA expression (transformed to log2 CDPV) was evaluated using the PCA function in GeneSpring.

### Network analysis

Co-expression analysis starts by constructing a matrix of pairwise correlations between all pairs of RNAs across all selected samples. As it is believed that low signal intensity candidates provide limited information in a co-expression network setting, candidates were selected based on their values (RPMK > 1 or TPM > 1, in 1 of the 9 samples), resulting in 16,903 mRNAs and 716 miRNAs. Consequently, we constructed an unsigned co-expression network using the WGCNA package^17^. Gene ontology (GO) term enrichment tests were performed for individual gene co-expression modules compared with a background set of all genes expressed in CD4^+^ T cells using the R packages GOstats (version 2.26.0), biomaRt version (2.14.0), AnnotationDbi (version 1.20.7) and org.Hs.eg.db (version 2.8.0). Human protein-protein associatome from STRING database was used to identify connected subnetworks from selected co-expression modules (highest confidence > 0.8).

To classify LL from other aging stages, unsupervised hierarchical clustering of the candidates in every trait module was performed by bootstrapping analysis using MeV software (http://www.tm4.org/). Bootstrapping analysis provides confidence values for the stability of each cluster derived by hierarchical clustering. Network hubs are defined as highly connected genes within a network, having high intramodular connectivity performed by VisANT software (http://visant.bu.edu/).

### WGCNA analysis

The adjacency matrix was created by calculating the Pearson’s correlations between all genes, and raised to a power β of 7. The power β was chosen based on the scale-free topology criterion, resulting in a scale-free topology index (R^2^) of 0.9. Next, the Topological Overlap Measure (TOM), representing the overlap in shared neighbors, was calculated using the adjacency matrix. Modules whose eigengenes are highly correlated are merged. The above steps were performed using the automatic network construction and module detection function (*blockwiseModules* in WGCNA), with the following major parameters: *minModuleSize* of 30, *reassignThreshold* of 0 and *mergeCutHeight* of 0.35. The dissimilarity TOM was used as input for the dendrogram, and modules (clusters of highly interconnected genes) were detected as branches of the dendrogram using the Dynamic Tree Cut algorithm. All modules were assigned to a color. The module eigengene was used to represent each module, which was calculated by the first principal component, thereby capturing the maximal amount of variation of the module. Using the module eigengene, the Module-Trait relationships were estimated by calculating the Pearson’s correlations between the module eigengene and the traits of interest. Those Module-Trait relationships were used to select potential biologically interesting modules for downstream analysis. Gene significance (GS): defined as GSi = |cor(xi, T)|, indicates correlation of a xi node expression profile to a phenotypic trait T, a binary trait variable across m samples. We used the *softconnectivity* function within the WGCNA package to calculate the connectivities of all genes in a network, which were then scaled to the maximum connectivity within that network. In this case, phenotypic trait is NB, MA and LL. To filter hubs significantly correlated to LL phenotype and identify a LL specific hub network, we used high values of GS, and intra-modular connectivity of top 100 candidates in every trait modules.

### Target prediction of miRNAs exhibiting different expression levels

We investigated the biological relevance of differentially expressed miRNAs via their regulation on target genes. First, putative microRNA-targeted genes were generated according to the intersection of the results from two prediction algorithms, miRanda 1.9 and TargetScan 6.2. Second, Pearson correlation analysis was performed using R software to determine whether the expression levels of each miRNA and its mRNA targets were negatively correlated (P-value < 0.05). Finally, the putative targets confirmed by the correlation analysis were subjected to further analysis.

### Identification of enriched methylated regions (peaks) and differentially methylated regions (DMRs)

An amount of 100–200 ng of DNA was isolated from CD4^+^ T cells from the NB, MA and LL groups using the AllPrep DNA Kit from Qiagen according to manufacturer’s instructions. MeDIP libraries of 150–200 bp were prepared for three biological replicates of the NB, MA and LL groups and were then subjected to 50 bp paired-end (PE) sequencing on the Illumina GA IIx platform. We generated averages of 106 M, 104 M and 106 M raw reads, resulting in 5.3, 5.2 and 5.3 raw Gb of paired-end sequence data from the NB, MA and LL groups processed through an Illumina GA IIx system, respectively. The saturation analysis indicated that we had sufficient reads to give reproducible genome-wide DNA methylation profiles (Supplementary Fig. S5C). Of these, averages of 4.4, 4.35 and 4.5 Gb (averages of 83%, 84% and 87% per group, respectively) were successfully aligned to either strand of the reference human genome (version Hg18, downloaded from http://hgdownload.cse.ucsc.edu/downloads), performed with SOAPaligner^39^ (Short Oligonucleotide Analysis Package, version 2.21) using -s 34 -v 2 -g 0 -r 1 -M 4 parameters. Reads mapped with SOAPaligner were associated with genes using the custom perl scripts that allowed no more than 2 unmapped bases. Only uniquely mapped reads were retained for subsequent analyses. To distinguish methylated DNA fragmentation, the so-called peaks, from the whole genome, we implemented this analysis using MACS (version 1.4.0)^40^ followed by the parameters with -gsize 10, 30 -pvalue 0.00001 -wig -wigextend 300 -space 5.

We employed a sliding-window method and rank-test to identify putative DMRs across the genome. Firstly, we linked two paired reads to one fragment. We then calculated the number of methylated reads in each 50-bp sub-bin of every 10-interval sliding window with a 250-bp step length for each sample. Rank-test was conducted in each sliding window to examine the significant differentially methylated windows (i.e. DMRs). One putative DMR met the following criteria: i) rank-test p-value less than 0.05; ii) the total count of reads in DMR for each sample more than 10; iii) at least one overlap of DMR and peak, 2-fold difference, and a significant difference (P < 0.05) using the chi-squared test.

After determining DMRs, we associated DMRs with gene elements and selected the genes of which the upstream, 5′-UTR and gene body overlapped DMRs. We then proceeded with functional analysis, searching for the functions and signaling pathways of the genes using known databases, including DAVID GO and Kyoto Encyclopedia of Genes and Genomes (KEGG) pathway (http://david.abcc.ncifcrf.gov/). Given the large number and complex branch structures of GO biological processes, we used a significance threshold P-value < 0.05 for biological process terms.

### Real-time quantitative polymerase chain reaction for mRNA and miRNA expression validation

Total RNA was isolated from CD4^+^ T cells using Trizol reagent (Qiagen, CA, USA). cDNA synthesis was performed using RevertAid™ First Strand cDNA Synthesis Kit (Fermentas, Burlington, Canada) and 1 μg of total RNA according to the manufacturer’s instructions. Real-time quantitative PCR was performed using a Rotor-Gene3000 (Corbett Research, NSW, Australia), and mRNA levels were quantified using SYBR Premix Ex Taq^TM^ real-time PCR Kit (TaKaRa Biotech [Dalian] Co., China). β-actin was also amplified and used as a loading control. The primers used are listed in Supplementary Table S7. miRNAs were detected using miRNA-specific primers and the miScript SYBR Green PCR Kit (Qiagen, Germany). U6 snRNA was used as a loading control for sample normalization. The relative levels of genes or miRNAs were calculated using the 2^−*Δ*Ct^ method: *Δ*Ct = Ct_Target gene or miRNA_ − Ct_β-actin or U6_.

### Bisulfite-based polymerase chain reaction for DNA methylation validation

Genomic DNA was isolated from CD4^+^ T cells using TIANamp Genomic DNA kit (Tiangen Biotech, Beijing, China). Bisulfite conversion was performed using the EpiTect Bisulfite Kit (Qiagen, Valencia, CA, USA) according to the manufacturer’s protocol. Promoter sequences with different DNA methylation patterns were amplified by PCR. PCR products were purified and cloned into pGEM-T vectors (Promega, Madison, WI, USA) and then transformed into E. coli cells. Ten positive recombinant clones were selected at random and sequenced using ABI 3730. Gene methylation levels were assessed according to the percentage of the methylated CG sites in ten clones. All primer sequences are listed in Supplementary Table S8.

### Statistical analysis

One-way ANOVAs were used to compare values of three groups. The Student’s *t*-test for equality of means was used to compare values between two groups. Data are presented as means ± standard deviations (SDs) and were analyzed using SPSS version 16.0 software (SPSS Inc., Chicago, IL, USA). Pearson’s correlation coefficient was used for the correlation analysis. P-values < 0.05 were considered as significant. Fisher’s exact test was used for the enrichment analysis with a cut off P-value < 0.05.

### Data Availability

Expression and DNA methylation data described in this study are available from Gene Expression Omnibus (GEO) database (www.ncbi.nlm.nih.gov/geo) with accession ID: GSE65515.

## Additional Information

**How to cite this article**: Zhao, M. *et al*. Distinct epigenomes in CD4^+^ T cells of newborns, middle-ages and centenarians. *Sci. Rep.* **6**, 38411; doi: 10.1038/srep38411 (2016).

**Publisher's note:** Springer Nature remains neutral with regard to jurisdictional claims in published maps and institutional affiliations.

## Supplementary Material

Supplementary Information

Supplementary Table S1

Supplementary Table S2

Supplementary Table S3

Supplementary Table S4

Supplementary Table S5

Supplementary Table S6

Supplementary Table S7

Supplementary Table S8
