# Supplementary material for: Distinct epigenomes in CD4+ T cells of newborns, middle-ages and centenarians
Source: Sci Rep. 2016 Dec 5;6:38411. doi: 10.1038/srep38411 (PMC5137168; doi:10.1038/srep38411)
Supplement: Supplementary Information [file srep38411-s1.pdf]

Distinct epigenomes in $CD4^+$ T cells of newborns, middle-ages and centenarians

Ming Zhao[1*], Jian Qin[2*], Hanqi Yin[3*], Yixin Tan[1], Wei Liao[1], Qian Liu[1], Shuangyan Luo[1], Min He[2], Gongping Liang[1], Yajing Shi[1], Qing Zhang[1], Wenjun Cai[4], Guangliang Yin[4], Yin Zhou[1], Jing Wang[1], Mengying Li[1], Yi Huang[1], Aiyun Liu[1], Haijing Wu[1], Zhiyong Zhang[2#], Qianjin Lu[1#]

[1]Department of Dermatology, The Second Xiangya Hospital, Central South University, Hunan Key Laboratory of Medical Epigenomics, Changsha 410011, PR. China. [2]Public Health College of Guangxi Medical University, Nanning 530021, PR. China. [3]State Key Laboratory of Biocontrol, Sun Yat-sen University, Guangzhou 510275, PR. China. [4]Beijing Genomics Institute at Shenzhen, Shenzhen 518083, P.R.China.

#Correspondence and requests for materials should be addressed to Z.Z. (rpazz@163.com) or to Q.L. (email: qianlu5860@gmail.com).

*These authors contributed equally to this work.

Supplementary Fig. S1. Volcano plots of differences in miRNAs and genes expression among NB, MA and LL. Differences in miRNAs and genes expression in CD4$^+$ T cells (n = 3). Each point represents a gene, with mean log 2 fold change along the x-axis and log10 of the P-value from DESeq package along the y-axis.

Supplementary Fig. S2. Genes expression of GLU, PLAG1, FCGR3B and GPR97 relative to internal control GAPDH, expression of miRNA-183-5p, miRNA-1537 and miRNA-106a-5p relative to internal control U6 were measured by qPCR in un-pooled samples including 6 NB samples, 8 MA samples and 6 LL samples. Data was shown as mean ± SD. *, P-value < 0.05, **, P-value < 0.01.

Supplementary Fig. S3. The mean methylation levels in promoter regions of ATP5G and RFWD2 in un-pooled samples, which were measured by bisulfite sequencing. (A) DNA methylation change of ATP5G gene between NB and MA samples. (B) DNA methylation change of RFWD2 gene between MA and LL samples. Data was shown as mean ± SD. **, P-value < 0.01.

Supplementary Fig. S4. Relative mRNA expression levels of BACH2 (A) and DNA methylation level of BACH2 promoter (B) were measured by RT-qPCR and BSP in CD4$^+$ T cells from NB, MA and LL individuals used for transcriptome-seq and methylome-seq. **, P-value < 0.01.

Supplementary Fig. S5. First single end reads from each sample were performed saturation analysis respectively, which indicated that we had sufficient reads to give reproducible genome-wide miRNA transcription, gene expression and DNA methylation profiles. (A) The saturation analysis indicates the reproducibility of the gene number given an increasing sequencing depth. (B) The saturation analysis indicates the reproducibility of the miRNA number given an increasing sequencing depth. (C) The saturation analysis with MEDIPS package indicates the reproducibility of the genome wide coverage at regular genomic intervals given an increasing sequencing

depth.

Supplementary Table S1. Candidates (Genes and non-coding RNA) and their expression levels in cluster 1-10.

Supplementary Table S2. Candidates (Genes and non-coding RNA) in blue module, royalblue module, darkorange module and turquoise module.

Supplementary Table S3. 7015 hypermethylated and 2820 hypomethylated DMRs in the NB compared with the MA (nmDMRs).

Supplementary Table S4. 4809 hypermethylated and 7553 hypomethylated DMRs in the MA compared with the LL (mlDMRs).

Supplementary Table S5. 636 significant aDMRs between MA and NB. 3595 significant aDMRs between MA and LL samples.

Supplementary Table S6. The proportions of naïve, memory and regulatory T cells, the values of genes and microRNAs expression and DNA methylation levels in the un-pooled samples used in the supplementary validation experiments, which were detected by flow cytometry, RT-qPCR and Bisulfite sequencing.

Supplementary Table S7. primers for RT-qPCR.

Supplementary Table S8. primers for BS-PCR.

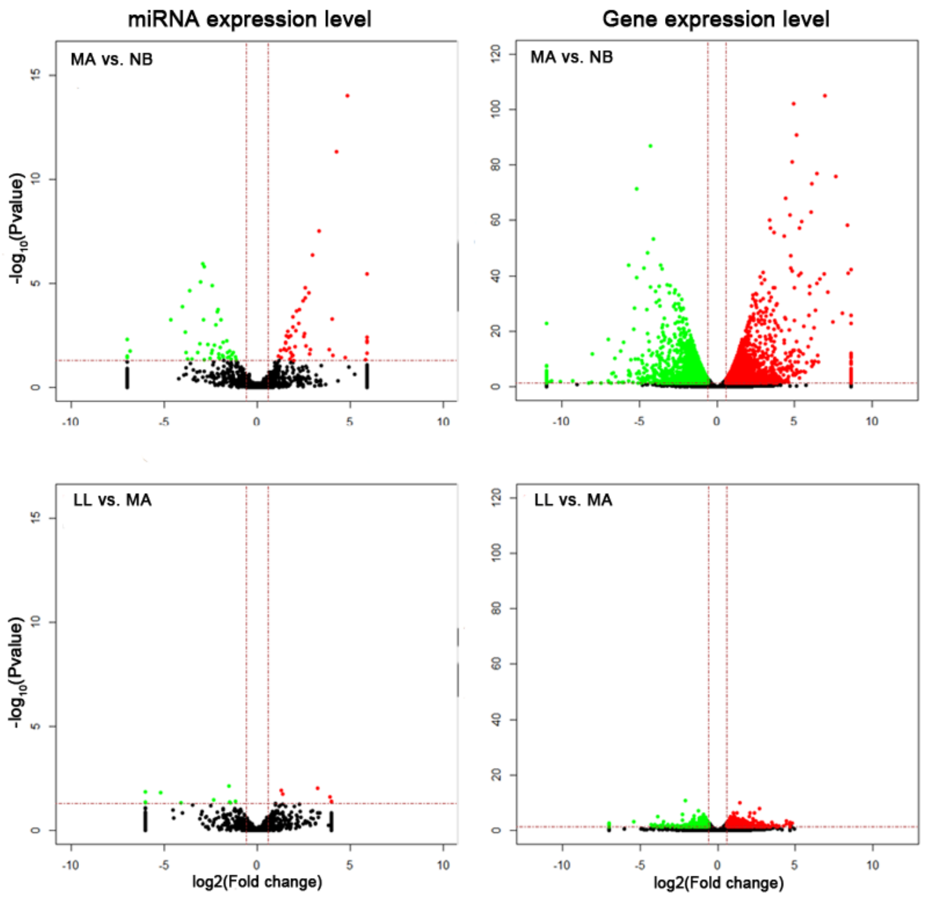

Supplementary Fig. S1

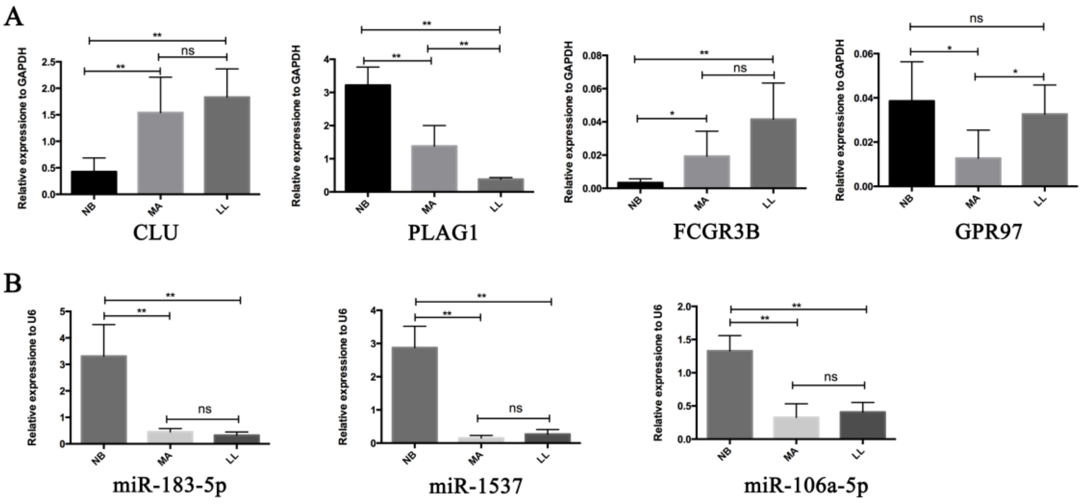

Supplementary Fig. S2

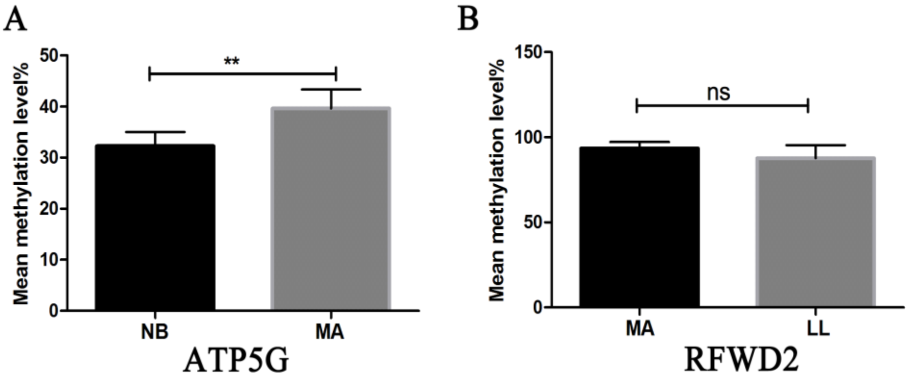

Supplementary Fig. S3

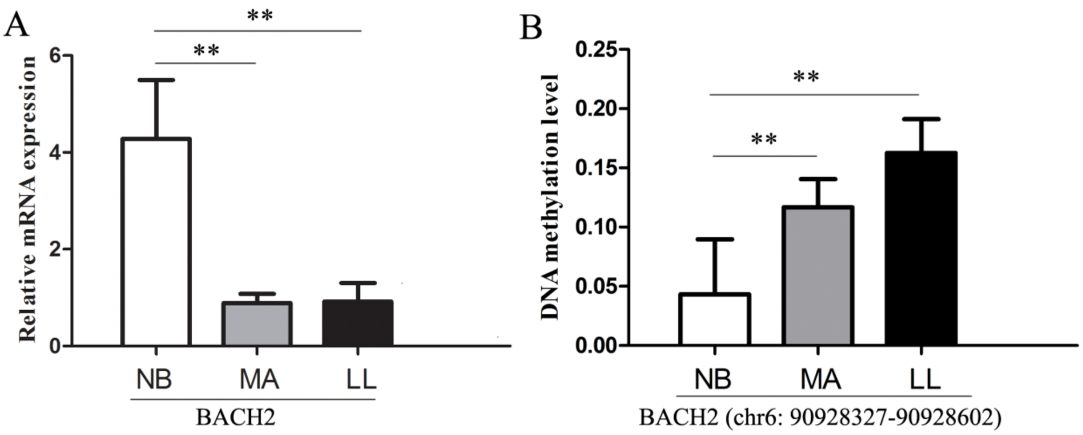

Supplementary Figure S4

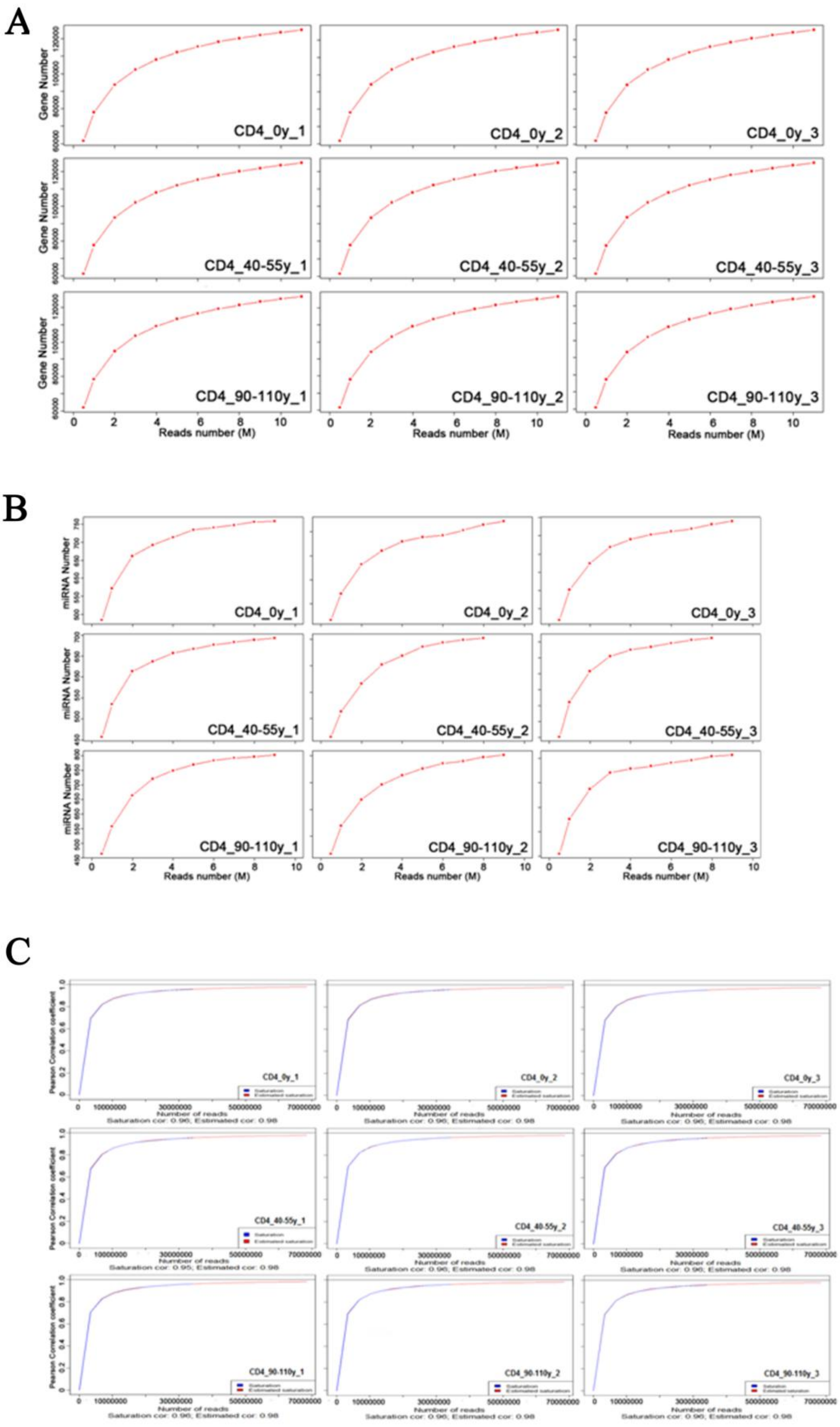

Supplementary Fig. S5
